# Dietary Heterogeneity among Western Industrialized Countries Reflected in the Stable Isotope Ratios of Human Hair

**DOI:** 10.1371/journal.pone.0034234

**Published:** 2012-03-30

**Authors:** Luciano O. Valenzuela, Lesley A. Chesson, Gabriel J. Bowen, Thure E. Cerling, James R. Ehleringer

**Affiliations:** 1 Department of Biology, University of Utah, Salt Lake City, Utah, United States of America; 2 IsoForensics, Inc., Salt Lake City, Utah, United States of America; 3 Department of Earth and Atmospheric Sciences, Purdue University, West Lafayette, Indiana, United States of America; 4 Department of Geology and Geophysics, University of Utah, Salt Lake City, Utah, United States of America; Institut Pluridisciplinaire Hubert Curien, France

## Abstract

Although the globalization of food production is often assumed to result in a homogenization of consumption patterns with a convergence towards a Western style diet, the resources used to make global food products may still be locally produced (*glocalization*). Stable isotope ratios of human hair can quantify the extent to which residents of industrialized nations have converged on a standardized diet or whether there is persistent heterogeneity and glocalization among countries as a result of different dietary patterns and the use of local food products. Here we report isotopic differences among carbon, nitrogen and sulfur isotope ratios of human hair collected in thirteen Western European countries and in the USA. European hair samples had significantly lower δ^13^C values (−22.7 to −18.3‰), and significantly higher δ^15^N (7.8 to 10.3‰) and δ^34^S (4.8 to 8.3‰) values than samples from the USA (δ^13^C: −21.9 to −15.0‰, δ^15^N: 6.7 to 9.9‰, δ^34^S: −1.2 to 9.9‰). Within Europe, we detected differences in hair δ^13^C and δ^34^S values among countries and covariation of isotope ratios with latitude and longitude. This geographic structuring of isotopic data suggests heterogeneity in the food resources used by citizens of industrialized nations and supports the presence of different dietary patterns within Western Europe despite globalization trends. Here we showed the potential of stable isotope analysis as a population-wide tool for dietary screening, particularly as a complement of dietary surveys, that can provide additional information on assimilated macronutrients and independent verification of data obtained by those self-reporting instruments.

## Introduction

The traditional view of the effects of globalization on food consumption is the convergence of dietary patterns across the globe [1]–[4]. This convergence is usually represented by a transition from region-specific dietary patterns towards a Western style diet high in animal products, vegetable oils and refined carbohydrates [1]–[4]. However, in recent years this view has changed to incorporate local influences on food production and culturally framed consumer choices [1], [4]–[6]. One outcome of this new understanding is the development of the concept of *glocalization*, which in the context of food production refers to the manufacturing of globally-distributed food items using local resources [5], [6]. For example, the McDonald's® Big Mac®, an icon of food globalization, has been recognized to be a *glocal* food item based on the geographic origin of its main component, beef [5]. By measuring stable carbon isotope ratios of beef Martinelli et al. [5] showed that the Big Mac® is produced in very much the same way across the globe but with locally or regionally produced beef. If a globalization symbol such as the Big Mac® indeed represents the use and consumption of local resources, then how global, *glocal* or local is the diet of modern industrialized societies? To what extents have the dietary origins of these societies converged and become homogeneous?

European countries are traditionally known to have regional dietary patterns driven by climate, geography and cultural differences [7], [8]. Nevertheless, modern industrialized European societies are undergoing dietary shifts towards a more-common Western style diet and are losing regional dietary characteristics [7], [9]–[14]. These shifts are particularly pronounced in adolescents and young adults, and are most noticeable in Nordic and Mediterranean countries [12], [13], [15]. Although changes are identifiable in today's European diet, it is difficult to determine if these dietary shifts entail changes in the origin of the resources consumed with an increase in the use of globalized resources.

Stable isotope analysis is becoming an increasingly useful tool for the study of human diet [16]–[25]. Carbon, nitrogen, and sulfur molecules in human tissues are derived solely from the diet, and their stable isotope ratios reflect those of consumed products. Stable carbon isotope ratios (δ^13^C) closely reflect the isotope ratios of the original dietary carbon source and have been used as indicators of the proportion of C_3_ (*e.g.*, temperate grasses, fruits, vegetables) or C_4_ (*e.g.*, tropical grasses, corn, sugar cane) plants in the human diet, either consumed directly as a staple food or indirectly as animal feed [17], [18], [26]–[28]. The stable nitrogen isotope ratios (δ^15^N) in the tissues of a consumer are generally enriched in ^15^N relative to diet [29]. Because of this enrichment, the δ^15^N values of analyzed tissues (e.g., hair, fingernails, bone) have been used as an indicator of the proportions of animal and plant proteins in the diet [20]–[23], [29]. Stable sulfur isotope ratios (δ^34^S) have primarily been used to differentiate inland diets from coastal diets that have a higher proportion of marine protein intake and thus higher δ^34^S values of ocean derived S-bearing molecules [25], [30]–[32]. Furthermore, the δ^34^S values of consumer tissues also correspond to geographic and geogenic variables such as atmospheric deposition as a function of distance to the coast (sea-spray effect) and the isotopic composition of the bedrock [30], [33], [34].

Human hair is a useful integrator of dietary information; its isotope ratios primarily reflect the proportional contributions of protein sources to the diet [23]. Consequently, the stable isotope ratios of human hair can be used to assess population trends and dietary differences across geographic areas. Cultural and geographic differences in diet have previously been detected in contemporary human groups by measuring isotope ratios in scalp hair and fingernail samples [18]–[25], [32], [35], [36]. The best-documented cases of dietary differences among industrialized countries detected with stable carbon isotopes of human hair are between American and British residents [20], [35], [36]. The clear isotopic separation between the two countries (with American hair samples having higher δ^13^C values than British samples) has been attributed to the widespread use of corn as feed for farm animals in the USA [20], [24], [35], [36]. Corn and other C_4_ plants on average have δ^13^C values approximately 14‰ higher than temperate grasses and other C_3_ plants (fruits and vegetables; [37]). However, with the exception of the studies on assignment of dietary preferences for residents of Germany conducted by Cornelia C. Metges, Klaus-Jürgen Petzke and many colleagues (see [23] for a review), limited isotopic data exist for modern hair samples of healthy individuals from industrialized European nations other than the United Kingdom (see also [38]).

We hypothesize that regional isotopic differences still persist in human hair from modern industrialized nations, despite recent dietary convergence, due to the influence of regional dietary patterns and the consumption of local and *glocal* products. We expect that southern countries will have higher δ^13^C values than northern countries given the global distribution of C_4_ and C_3_ plants [39] and their influence on locally produced meats, as detected by Martinelli et al. [5] for the Big Mac®. We also predict regional differences in the δ^34^S values of hair given the known higher consumption of marine resources within the Iberian Peninsula in comparison to other European countries [40], [41]. Furthermore, we expect no differences in δ^15^N values given the high prevalence of animal proteins (derived from herbivores, such as cows) in the diet of most modern Europeans. Here we present novel data on carbon, nitrogen and sulfur isotope ratios for human hair collected across thirteen European countries. In addition, we also compare the isotope ratios of European hair with a published USA dataset [33] to test whether the previously observed isotopic difference between the USA and England [20], [35], [36] is also observed in other industrialized nations across Europe.

## Methods

### Ethics statement

This research was approved by the Institutional Review Board (IRB) of the University of Utah under protocol number 10249.

### Sample collection and processing

**Figure 1:**
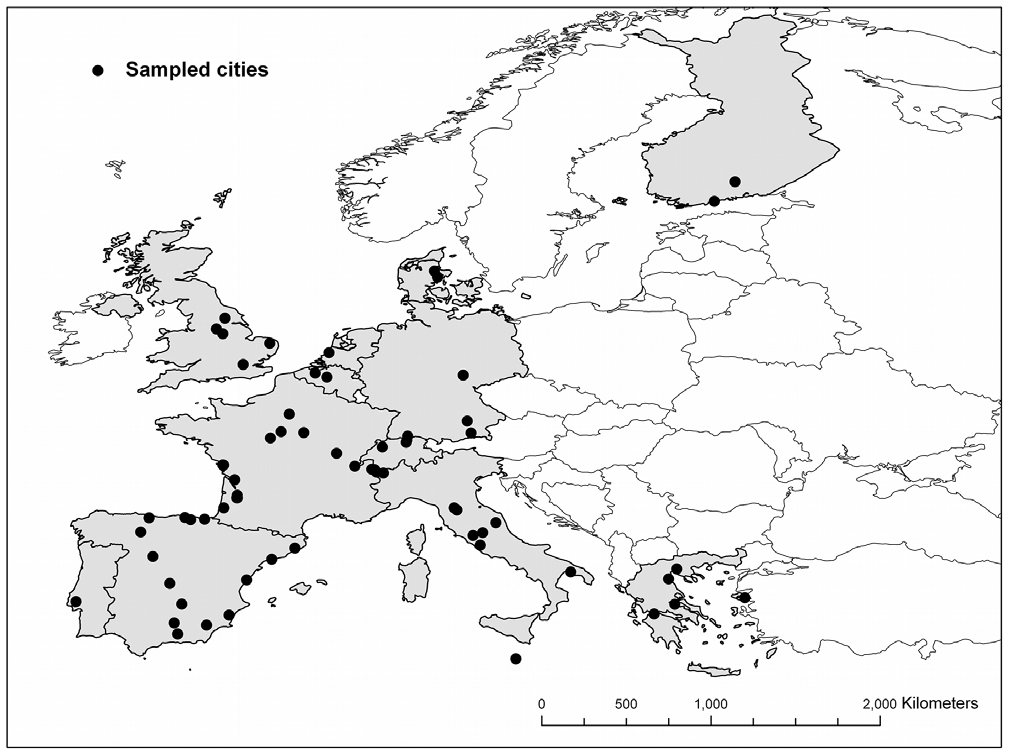
Distribution of sample locations in Western Europe. Countries where samples were collected are highlighted in grey; black circles represent cities where sampled were obtained. For the names of the cities see Table S1. For the distribution of sample locations in the USA see [33].

Human scalp hair was collected as trash from the floors of barbershops and donated by anonymous volunteers in thirteen Western European (WE) countries (Figure 1; Table S1). Although we use the term ‘Western Europe’ to group all thirteen countries sampled in our study, we recognize that some (e.g., Greece, Italy and Malta) are not always classified as Western Europe but rather as Southern Europe. For the United States of America (USA) we used the same dataset of hair samples described by Valenzuela et al. [33], with the addition of 28 new samples. These 28 new samples did not represent new collection sites and thus the sampling locations are the same as those presented in Figure 1 in Valenzuela et al. [33]. All hair samples were placed in paper envelopes at the time of collection. No information was recorded regarding the age, gender, diet, and health or travel history of the donors. We assumed that the hair samples represented individuals local to the collection site. Prior to analysis, hair samples consisting of 20–40 strands of hair were washed twice in a 2∶1 chloroform:methanol mixture at room temperature to remove lipids and other surface contaminants. In the case of dyed hair, the washes were repeated until the solvent mixture was clear and no additional color was leached from the hair. The volume of solvent mixture used in each wash was sufficient to completely submerge all hair strands. The solvent mixture was gently agitated during the washes. After the washes were completed the samples were placed in paper filters and left to dry inside a fume hood. Once the samples were dried they were ground to a fine powder using a ball mill (Retsch; Haan, Germany) and placed in capped 1-dram glass vials for storage until analysis. For δ^13^C and δ^15^N analysis, 500 µg (±10%) of ground material was loaded into tin capsules (3.5×5 mm, Costech Analytical; Valencia, CA, USA); for δ^34^S analysis, 900 µg (±10%) was loaded into tin capsules.

### Stable isotope analyses

Hair samples were analyzed using an isotope ratio mass spectrometer (IRMS) operated in continuous flow mode (Finnigan-MAT Delta S; Bremen, Germany). Analyses of hair samples for combined carbon and nitrogen as well as for sulfur isotopes were conducted using different instruments, but the analytical procedures were similar. Tin capsules were loaded into a zero-blank autosampler (Costech Analytical, Valencia, CA, USA) interfaced with an elemental analyzer (Carlo Erba, Milan, Italy) where they were flash combusted to produce CO_2_, N_2_, and SO_2_ for carbon, nitrogen, and sulfur isotope analysis, respectively. To minimize the need for oxygen isotope corrections of the SO_2_ gas we followed the method developed by Fry et al. [42]. The resulting gases were chromatographically purified and carried to the IRMS via He stream. For δ^13^C and δ^15^N analysis a 3-m Poraplot-Q packed gas chromatography column (80 mL min^−1^, 40°C) was used, and for δ^34^S analysis a 0.8-m Hayes Sep packed gas chromatography column (80 mL min^−1^, 90°C) was used. Hair samples were analyzed alongside a set of internal laboratory reference materials. For carbon and nitrogen analyses, the isotope ratios of our laboratory reference material, Powdered Queratin (Voigt Global Distribution Inc., Lawrence, KS, USA), had values of −24.04‰ for δ^13^C, +5.93‰ for δ^15^N when calibrated against the international standards USGS40 (−26.389‰ for δ^13^C, −4.5‰ for δ^15^N) and USGS41 (+37.626‰ for δ^13^C, +47.6‰ for δ^15^N). For sulfur analyses, the isotope ratios of our laboratory reference materials, silver sulfide (Salt Lake Metals, Salt Lake City, UT, USA), zinc sulfide (Mass Spectrometry Laboratory, Institute of Physics, Marie Curie-Sklodowska University, Poland) and powdered feathers (International Down and Feather Testing Laboratory, Salt Lake City, UT, USA), had values of +17.94, −31.94 and +16.9‰, respectively, when calibrated against the international standards IAEA-S-1, 2 and 3 (−0.30, +22.7 and −32.3‰, respectively). Results for δ^13^C values are presented on the Vienna Pee Dee Belemnite (VPDB) scale; for δ^15^N values, on the atmospheric AIR scale; and for δ^34^S values, on the Vienna Canyon Diablo Troilite (VCDT) scale. The analytical precision (1σ) based on long term measurements of internal laboratory reference materials for δ^13^C, δ^15^N and δ^34^S values was 0.1‰, 0.2‰ and 0.4‰, respectively. Stable isotope ratios are reported following new guidelines by Coplen [43] and using the standard δ-notation relative to an international standard in units per thousand (‰) as follows: δ = (R_sample_/R_standard_ − 1), where R_sample_ and R_standard_ are the molar ratios of the heavy to light isotopes (e.g., ^13^C/^12^C) of the sample and standard, respectively.

### Statistical analyses

Data are presented as means ±1 standard deviation unless stated otherwise. Normality and homoscedasticity were tested with Shapiro-Wilk and Bartlett's tests, respectively. Unpaired t-tests were used to compare stable isotope values of samples from Europe and from the USA. Single-classification Analyses of Variance (ANOVA) was used to compare isotope ratios among European countries. Due to the covariation of latitude with longitude in the European dataset (Figure 1), we used partial correlation analysis to assess the correlation of isotope ratios with latitude, or longitude, while controlling for the effect of longitude, or latitude respectively; for example, the correlation coefficient of δ^13^C values and latitude controlling for longitude is expressed as *r_CLat.Long_*. Data analysis and statistical calculations were conducted using R [44]. Statistical significance was set at α = 0.05.

## Results

**Figure 2:**
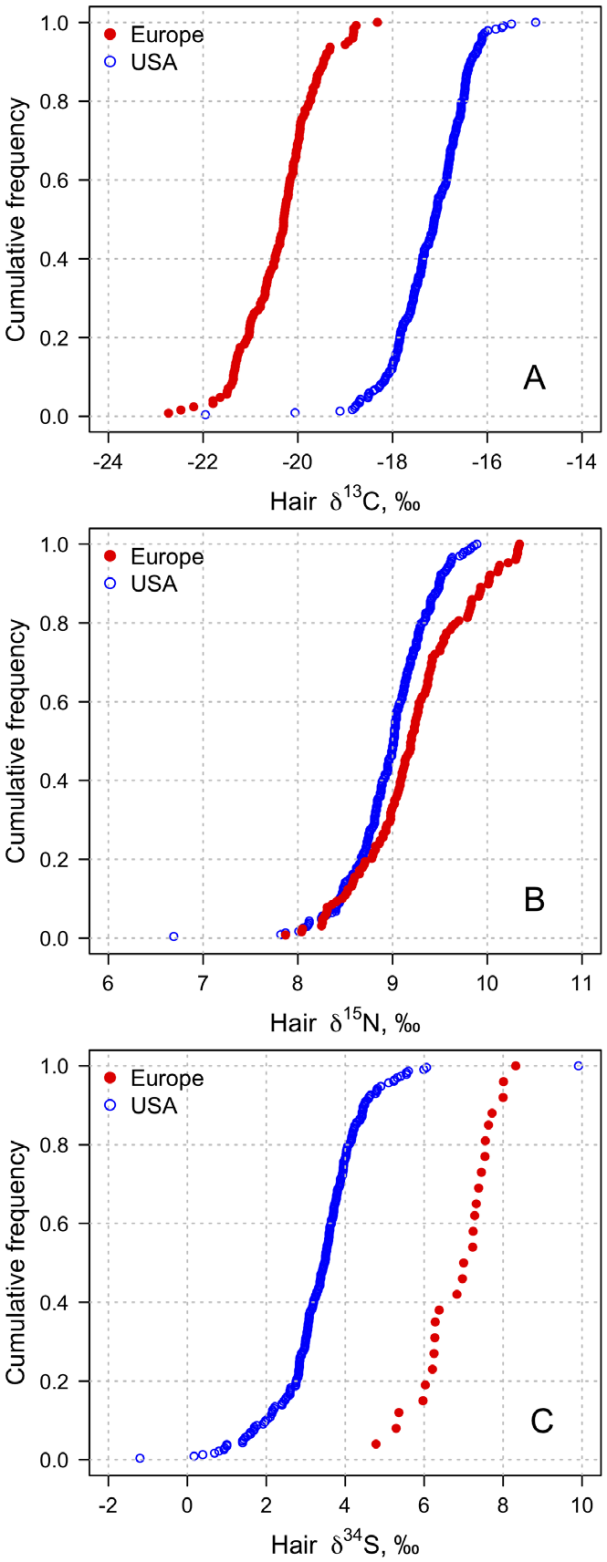
Cumulative frequencies for measured A) δ^13^C, B) δ^15^N and C) δ^34^S values of hair samples collected in Europe (filled circles) and the USA (open circles). Note the outlier in the USA distributions; the same sample had the lowest δ^13^C and δ^15^N values and the highest δ^34^S value. Western Europe ranges: δ^13^C: −22.7 to −18.3‰, δ^15^N: 7.8 to 10.3‰, δ^34^S: 4.8 to 8.3‰. USA ranges: δ^13^C −21.9 to −15.0, δ^15^N: 6.7 to 9.9‰, δ^34^S: −1.2 to 9.9‰.

The ranges of carbon, nitrogen and sulfur isotope values in human hair collected across thirteen Western European countries were −22.7 to −18.3‰, 7.8 to 10.3‰ and 4.8 to 8.3‰, respectively (Figure 2). Descriptive statistics for the measured δ^13^C, δ^15^N and δ^34^S values by country are summarized in Table 1. No significant correlations were detected between stable isotope ratios of different elements (δ^13^C vs. δ^15^N, δ^13^C vs. δ^34^S, δ^15^N vs. δ^34^S) for samples collected in Western Europe (*p*>0.1, *r* ^2^≤0.1 for the three comparisons).

**Table 1:** Carbon, nitrogen and sulfur isotope values for hair samples collected in thirteen European countries and in the USA.

Country	δ^13^C, ‰	δ^15^N, ‰	δ^34^S, ‰
Belgium	−20.9±0.3 (3)	8.3±0.2 (3)	—
Denmark	−21.4±0.0 (2)	9.0±0.1 (2)	—
England	−21.1±0.5 (21)^a^	9.2±0.4 (22)^c^	—
Finland	−22.0±0.6 (3)	10.1±0.3 (3)	5.9±0.6 (5)
France	−20.0±0.7 (28)^b^	9.1±0.5 (29)^c^	7.5±0.4 (5)
Germany	−20.9±0.2 (4)	8.5±0.6 (4)	6.8±0.5 (4)
Greece	−21.0±0.3 (5)	8.9±0.5 (5)	—
Italy	−20.1±0.3 (13)^b^	9.2±0.5 (13)^c^	6.6±1.3 (4)
Malta	−20.1 (1)	9.9 (1)	—
Netherlands	−21.0 (1)	9.4 (1)	7.0 (1)
Portugal	−18.8 (1)	9.9 (1)	—
Spain	−19.9±0.7 (40)^b^	9.4±0.6 (41)^c^	7.3±0.7 (7)
Switzerland	−20.9±0.6 (4)	9.0±0.2 (4)	—
Western Europe	−20.3±0.8 (126)	9.2±0.5 (129)	6.8±0.9 (26)
United States of America*	−17.2±0.8 (234)	8.9±0.4 (234)	3.4±1.1 (228)

* Includes data previously presented in [33].

Isotope ratios are presented as mean ± standard deviation (sample size). δ^13^C and δ^15^N values for England, France, Italy and Spain were compared by single-classification ANOVA and Tukey HSD (Tukey-Kramer) pairwise post-hoc tests. Countries with the same letter were not statistically different (*p*<0.0001).

Hair collected across Western Europe and across the USA showed significantly different stable carbon, nitrogen and sulfur isotope ratios (Table 1, Figure 2). Hair from Europe had lower δ^13^C values (−20.3±0.8‰, n = 126) than hair from the USA (−17.2±0.8‰, n = 234; t-test *t* = 34.53, *p*<0.001; Figure 2A). The nitrogen isotope ratios of European hair (9.2±0.5‰, n = 129) were statistically higher (*t* = 4.67, *p*<0.001) than the values of hair from the USA (8.9±0.4‰, n = 234; Figure 2B). The sulfur isotope ratios of European samples (6.9±0.9‰, n = 26) were also significantly higher (*t* = 18.06, *p*<0.001) than the values of samples from the USA (3.4±1.1‰, n = 228, Figure 2C).

The ANOVA among European countries with more than 10 hair samples (England, France, Italy and Spain; Table 1) revealed significant differences for δ^13^C values (*F* = 16.8, *p*<0.001, n = 102) but not for δ^15^N values (*F* = 1.7, *p* = 0.2, n = 102). *Post-hoc* tests showed that England had significantly lower δ^13^C values than France, Italy and Spain (Tukey HSD test, *p*<0.001; Table 1). A parametric ANOVA could not be conducted for the measured δ^34^S values due to small sample sizes (Table 1); consequently, a non-parametric Kruskal-Wallis analysis of variance was used to test for differences among countries. The Kruskal-Wallis test reported significant differences among countries (K-W *χ* ^2^ = 10.8, *p* = 0.03, n = 26); with Spain and France typically reporting higher δ^34^S values, England and Italy middle values, and Finland lower values (Table 1; no *post-hoc* test was conducted).

**Figure 3:**
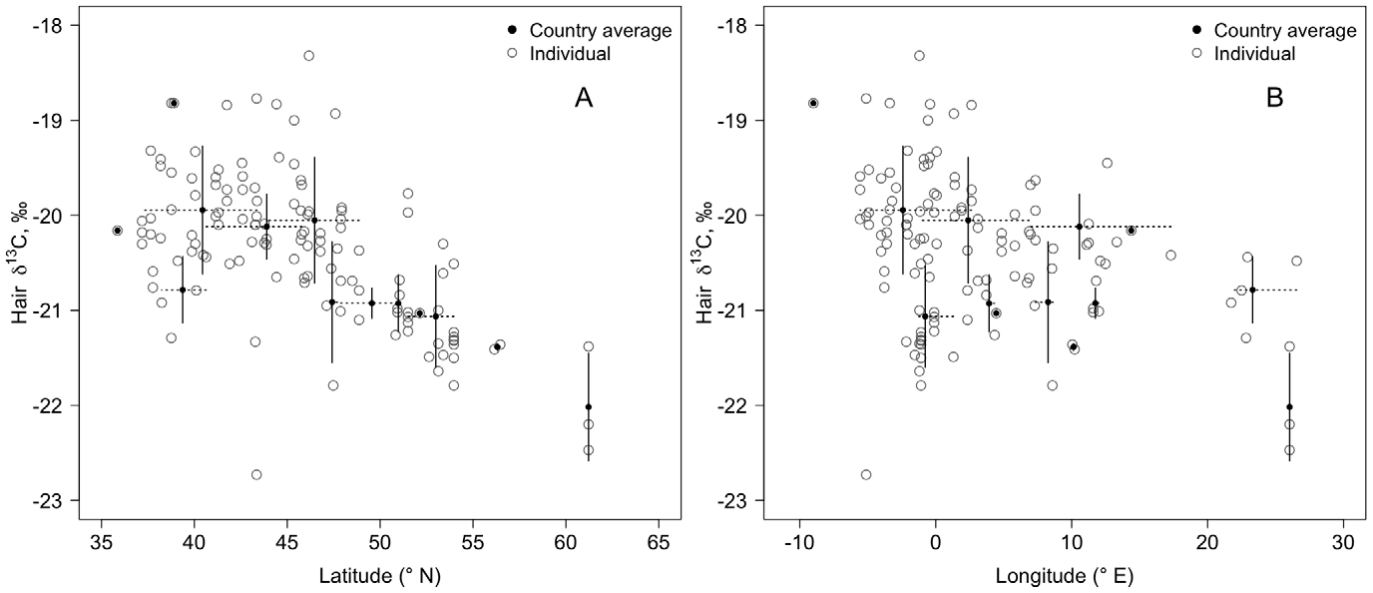
Covariation of δ^13^C values of human hair with A) latitude and B) longitude for samples collected in Europe. Open circles are individual hair samples and filled circles represent country averages (see Table 1). Solid lines represent the standard deviations of δ^13^C values by country. Horizontal dashed lines represent the latitudinal (A) or longitudinal (B) ranges covered during sample collection per country. Location means were estimated as the average latitude (A) or longitude (B) for collected samples per country. Partial correlations were significant (*r_CLat.Long_* = −0.56 (A), *r_CLong.Lat_* = −0.27 (B), *p*<0.001).

**Figure 4:**
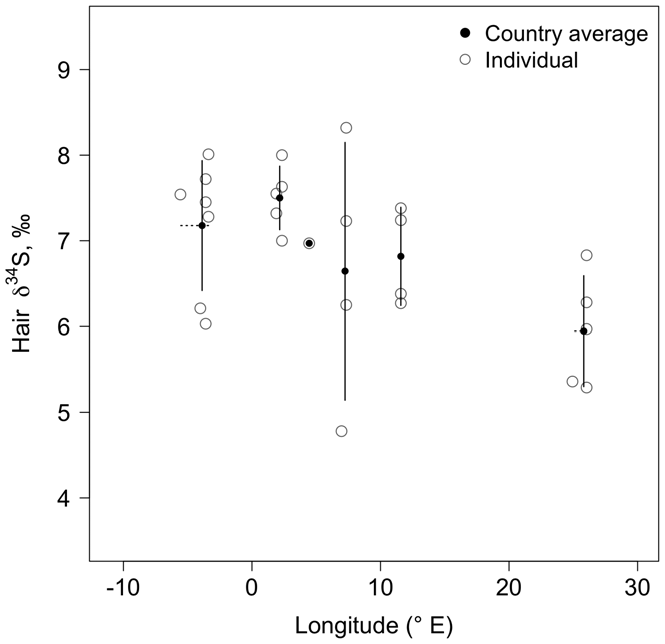
Covariation of δ^34^S values of human hair and longitude for samples collected in Europe. Open circles are individual hair samples; filled circles represent country averages (see Table 1). Solid lines represent the standard deviations of δ^34^S values by country. Horizontal dashed lines represent the longitudinal range covered by the samples analyzed per country. Location means were estimated as the average longitude for collected samples per country. Partial correlation controlling for latitude was significant (*r_SLong.Lat_* = −0.40, *p* = 0.03).

Within Western Europe, the δ^13^C values of human hair were negatively correlated with latitude (partial correlation *r_CLat.Long_* = −0.56, *p*<0.001, n = 126; Figure 3A) and longitude (*r_CLong.Lat_* = −0.27, *p*<0.01, n = 126; Figure 3B). No significant correlations were detected between the δ^15^N values and latitude (*r_NLat.Long_* = −0.02, *p* = 0.82, n = 129) or longitude (*r_NLong.Lat_* = −0.10, *p* = 0.26, n = 129). Sulfur isotope values were not correlated with latitude (*r_SLat.Long_* = −0.22, *p* = 0.26, n = 26) but were negatively correlated with longitude (*r_SLong.Lat_* = −0.40, *p* = 0.03, n = 26; Figure 4). However, this latter correlation is driven by the lower hair values of Finland (Figure 4); if this country is excluded from the analysis the correlation is not significant (*r_SLong.Lat_* = −0.31, *p* = 0.15, n = 21).

## Discussion

### Isotope variability of human hair from industrialized nations reflects dietary heterogeneity

As predicted, we observed isotopic differences in human scalp hair among thirteen western industrialized countries of Europe and the USA. We have assumed that the hair samples belonged to healthy individuals without metabolic or nutritional diseases that might influence their stable isotope ratios of hair protein [45]–[48]. Thus, we propose that the isotopic heterogeneity detected in this study is a result of dietary heterogeneity among the sampled regions because the stable carbon, nitrogen and sulfur isotope ratios in hair from healthy individuals are primarily determined by the isotopic composition of the protein sources and the relative consumption of those proteins [23].

The stable carbon, nitrogen and sulfur isotope ratios presented in our study are consistent with values previously published in smaller datasets of hair and fingernail isotope ratios for a few European countries [20]–[22], [24], [35], [38]. The absolute ranges of carbon (4.5‰), nitrogen (2.5‰) and sulfur (3.5‰) values calculated for the European dataset were smaller than for USA hair samples (6.9‰, 3.2‰ and 11.1‰ for δ^13^C, δ^15^N and δ^34^S values, respectively; Figure 2). However, the USA dataset contains one particular sample that can be considered an outlier (Figure 2; a single sample had the lowest δ^13^C and δ^15^N values, and the highest δ^34^S value). That outlier was from a sample obtained in Chicago, IL and it is possible that this individual could have been a visitor. When this outlier was removed the ranges for carbon (4.3‰) and nitrogen (2.2‰) isotopes in the USA dataset were similar to the European dataset. This suggested that the carbon and nitrogen isotopic ranges of consumed protein were likely similar in the two regions. The range in δ^34^S values in the USA remained approximately twice (6.5‰) that of the European samples when the outlier was removed, although the sample size for Western Europe was substantially smaller than for the USA (n = 26 vs. 228).

Overall, most samples from Western European countries had lower δ^13^C values than samples from the USA. The overlap in the distributions (between −19.3 and −18.3‰; Figure 2A) was represented by only eight samples from southwestern Europe (Blois, Bordeaux, Laleu, and Mirambeau from France; Cangas de Onis, Hostalric, and Valdepenas from Spain; and Almada from Portugal). This segregation in carbon isotope values of modern human hair between Western Europe and the USA is consistent with limited datasets presented in previously published work [20], [24], [35], [36]. Here we have shown with a larger dataset (total n = 360) that the isotopic separation remained significant, including observations from thirteen different European countries and 25 states across the USA. This stable isotope ratio difference has been attributed to the consumption of animal protein raised primarily with corn silage in the USA and with C_3_ silage and pasture in Europe [24], [35]. The widespread use of corn fodder in the USA contrasted with a much lower use of this crop in the European farms is evident in the carbon isotope ratio of meats from these regions published to date [5], [49]–[54].

Within Europe, a negative correlation between hair δ^13^C values and latitude exists and the trend agrees with our prediction. That is, the pattern is consistent with the regional decreases in the proportion of C_4_ grasses from the tropics towards higher latitudes [39]. If consumed animal proteins were of local origin and grown on local food stocks, then we would expect that the carbon isotope ratios of the consumer eating these proteins would also follow this latitudinal trend. Accordingly, this suggests consumption of locally or regionally produced foodstuff throughout Western Europe. A similar pattern was observed for the beef used in the Big Mac® worldwide by Martinelli et al. [5] where higher δ^13^C values were associated with lower latitudes and lower δ^13^C values towards higher latitudes. In addition, the higher δ^13^C values observed in the European south (particularly in the Iberian Peninsula) could be influenced by higher consumption of marine resources with high δ^13^C values in these countries. This would also explain the weak but significant correlation of δ^13^C values and longitude observed in Western European hair. However, this trend seems to be primarily driven by the low δ^13^C values of Finnish samples (Figure 3B).

The stable nitrogen isotope distributions of Western Europe and USA hair samples are characterized by extensive overlap; all samples from the USA (except the outlier at 6.7‰) were contained within the European range. The similarities in these continental distributions and the lack of geographic trends within Western European countries suggested 1) that residents of these countries consume similar amounts of animal protein, including dietary styles from vegans with no animal protein intake to omnivores with high animal protein intake [21], [22], and 2) that the baseline δ^15^N value of crops and animal feed may be similar [54]–[58]. The small number of higher δ^15^N values detected in Western Europe did not originate from a single geographic region; rather, they came from six different countries (Heinola, Finland; Mirambeau, France; Aosta, Italy; Dingli, Malta; Almada, Portugal; Medina del Campo, Cangas de Onis, Hostalric, Bilbao, and Toledo, Spain; and Sheffield, England). These samples might represent individuals with diets with unusually high animal protein consumption, consumption of elevated amounts of marine fish, or consumption of foodstuff derived from organic farming where manure is used as fertilizer [59].

The difference in the δ^34^S values between hair samples from Western Europe and the USA was interesting. Previous work with modern European hair samples is limited to a few samples from England [35]; accordingly, we had no prior expectations. The factors affecting δ^34^S values in humans are dependent upon the source of proteins being consumed. Marine proteins (e.g., fish, shellfish) have on average higher δ^34^S values than terrestrial proteins [60]. Within terrestrial agro-ecosystems, geographic location and farming practices may further influence the δ^34^S values of foods produced. Plants, and the animals consuming them, grown near the coastline tend to have high δ^34^S values due to the deposition and assimilation of sulfur molecules carried from the ocean by the wind (sea spray effect; [30]). However, a recent study showed that the influence of marine sulfur inputs on terrestrial animals was very localized (<100 km from the coastline; [34]). Furthermore, the soils where plants grow may have different δ^34^S values according to geologic origin [30]. Similarly, the type of fertilizers used will impact the isotope values of the crops. Large ranges in δ^34^S values (up to 30‰) of fertilizers exist due to their varied geochemical origins and manufacturing methods [59]. Given that all these processes could be acting at different geographic scales alone or in conjunction, it is very difficult to ascribe a single factor or combination of factors that would explain the isotopic difference in δ^34^S values of hair from Western Europe and the USA. Although the sample sizes for δ^34^S values of European countries are too small for rigorous interpretation in such a large geographic area, the differences among countries and the potential longitudinal trend were consistent with two hypotheses: 1) higher consumption of marine resources in Spain and France than in Germany and Italy [41], and 2) higher marine sulfur deposition on the western European coasts [34]. These differences are well reflected in the δ^13^C and δ^34^S values for the two areas presented in Figures 2A and 2C.

### Hair from Western European citizens reflects the existing regional dietary heterogeneity

Western Europeans and Americans consume large amounts of protein, in most cases in excess of the average nutritional requirements [41], [61]–[63]. However, agrofood industry practices and individual dietary choices based on cultural differences and food availability can have dramatic effects on the type of protein that is consumed in these regions, and therefore on hair isotope ratios, as observed in our study. The two main differences between Western Europe and the USA are 1) the pervasive use of corn in the American diet [64], and 2) the higher proportion of marine resources in the European diet [41], [65].

Dietary heterogeneity within Western Europe has been previously assessed through the use of dietary surveys such as food frequency questionnaires and 24-hour recall as well as the use of data from the Food and Agriculture Organization of the United Nations Statistical Division (FAOSTAT) [7]–[13], [40], [41], [63], [66]–[68]. The European Prospective Investigation into Cancer and Nutrition (EPIC) project in particular has provided great insights into the nutrition and dietary patterns of Western Europe in recent years [69] (http://epic.iarc.fr). The EPIC studies describe large levels of heterogeneity in foodstuff, beverages and overall dietary patterns between and within countries [8], [40], [63], [66], [68]. Generally, eastern Mediterranean countries such as Italy and Greece show the lowest numbers of food groups consumed, while France and Spain show the most heterogeneous diet [8], [66]. Western European citizens are characterized as high animal-protein consumers, with animal sources contributing more than 50% of total protein intake (in some cases more than 70%) [63]. Although red meat is the most prominent animal protein consumed across Europe, the relative contributions from other meats differ among countries [63]. For example, poultry is an important source of protein in England (∼20% of mean animal protein) but a very small one in Nordic countries (< than 5%) [8], [63]. Consumption of processed meats (e.g., sausages) is relatively high in Germany (25–30%) but very low in Greece [8], [63]. Spain, Greece and the Nordic countries are characterized by relatively high (∼20%) consumption of fish and other marine resources, while Germany and the Netherlands report very low relative intakes [8], [40], [41], [63]. In the case of Finland (sampled in our study but not part of the EPIC project), there is evidence that freshwater fish rather than marine fish represent a very large proportion of the everyday diet [65], [70].

The proportional contribution of different plant proteins to the average Western European diet also presents marked geographic differences [8], [66]. Although most plant protein sources consumed by Europeans are C_3_ plants (potatoes, vegetables, fruits and most cereals and legumes), the geographic differences in their relative intake are depicted in the heterogeneity of European dietary patterns [8], [66]. As a proportion of total plant protein, cereal (the overall highest contributor to plant protein) intake is higher in Mediterranean countries and in Scandinavian countries, while Spain and Germany report lower intake [63], [66]. Furthermore, there are clear south-north increasing trends in the relative consumption of legumes, fruits and vegetables [63], [66].

The dietary heterogeneity discussed above for Western Europe represents a complex set of regional and cultural differences affecting the consumption patterns of local residents. Thus, it is difficult to compare directly these dietary patterns and the isotopic data. We present two reasons for this difficulty: 1) The number of samples in our study is small in comparison to the thousands of individuals interviewed in efforts such as the EPIC project [69]; 2) The stable isotopic compositions of a consumer's tissues reflect the proportional contribution of all dietary sources to that tissue and different combinations of sources could produce similar isotope results. However, it is possible to observe some dietary trends reflected in both dietary patterns and isotopic data. The measured isotopic compositions of hair tissues from Western Europe tend to reflect dietary differences in cases where a “food group” is overwhelmingly represented in some regions and is characterized by very low intake in other regions. For example, residents of the Iberian Peninsula (and some regions of France) typically have a higher consumption of animal protein, particularly a much higher intake of seafood and marine fish, than other European countries, which coincides well with higher δ^13^C and δ^34^S values (and slightly higher – but not statistically different – δ^15^N values) observed for hair samples collected in these regions. It is also interesting to note that some of the lowest δ^13^C and δ^34^S values and some of the highest δ^15^N values were observed for Finnish samples, which coincides well with the idea that freshwater fish represents a large proportion of the protein consumed in Finland.

Even though globalization processes are acting at every level of the modern human food web separating people from their local resources (e.g., homogenization and industrialization of farming practices, large-scale distribution of food products and raw materials), here we have shown that regional dietary signatures are reflected in the tissues of consumers. Despite homogenization trends already detected in Europe [7], [11]–[14], isotopic data of modern human hair from Western Europe support the existence of dietary heterogeneity, which has characterized European observations for many years [7], [8], [69]. While isotope data cannot fully resolve whether these patterns reflect consumers eating different locally produced foods or eating the same food items produced with local resources (*glocal* food), they capture a geographically structured heterogeneous diet among the sampled countries.

Here we showed the potential that isotope analyses have for large-scale, rapid and efficient dietary screening. Stable isotope analyses will certainly not replace the level of detail generated with well-designed dietary questionnaires; rather they will complement those surveys and provide additional information on assimilated macronutrients. Stable isotope analyses could even provide independent verification of data obtained by self-reporting instruments. Furthermore, stable isotope analysis of hair could be accompanied with measurements of carbon isotopes in breath CO_2_ (as biomarker of sweetener consumption) to provide a broader view of dietary patterns across large regions [51], [71].

## Supporting Information

Table S1 **Cities where hair samples were collected in Western Europe.**
(PDF)
