# Supplementary material for: Dietary Heterogeneity among Western Industrialized Countries Reflected in the Stable Isotope Ratios of Human Hair
Source: PLoS One. 2012 Mar 30;7(3):e34234. doi: 10.1371/journal.pone.0034234 (PMC3316624; doi:10.1371/journal.pone.0034234)
Supplement: Table S1 — Cities where hair samples were collected in Western Europe. (PDF) [file pone.0034234.s001.pdf]

Country	City	Latitude (° N)	Longitude (° E)	Population
Belgium	Brussels	50.833	4.333	1830000
Belgium	Ghent	51.050	3.717	243366
Denmark	Aarhus	56.150	10.217	249709
Denmark	Randers	56.467	10.050	60656
England	London	51.500	-0.117	7825200
England	Mansfield	53.133	-1.200	67885
England	Norwich	52.633	1.300	259100
England	Sheffield	53.369	-1.540	555500
England	York	53.967	-1.083	202400
Finland	Heinola	61.217	26.033	20259
Finland	Helsinki	60.176	24.934	588941
France	Bellegarde-sur-Valserine	46.100	5.817	11404
France	Blois	47.591	1.335	47854
France	Bordeaux	44.420	-0.431	235891
France	Castets	43.883	-1.147	1885
France	Chalon-sur-Saone	46.783	4.850	48376
France	Chamonix	45.917	6.867	9514
France	Charny	47.883	3.100	1716
France	Chedde	45.933	6.717	10996
France	La Rochelle	46.164	-1.152	75822
France	Laleu	46.169	-1.200	90575
France	Mirambeau	45.373	-0.571	1486
France	Orleans	47.930	1.896	116490
France	Paris	48.867	2.333	12089098
Germany	Bad Aibling	47.867	12.000	18272
Germany	Jena	50.933	11.583	105129
Germany	Oberappersdorf	48.500	11.800	4271
Greece	Agios Konstantinos	38.758	22.830	3410
Greece	Litochoro	40.104	22.500	7011
Greece	Mytilini	39.110	26.555	36196
Greece	Patras	38.244	21.734	202757
Greece	Thessaloniki	40.635	22.936	1006730
Italy	Aosta	45.733	7.333	35061
Italy	Courmayeur	45.783	6.967	2870
Italy	Florence	43.767	11.250	370702
Italy	Prato	43.883	11.100	186710
Italy	Rome	41.900	12.483	2761477
Italy	San Ginesio	43.100	13.317	3872
Italy	Taranto	40.476	17.300	191810
Italy	Terni	42.567	12.617	112253
Italy	Viterbo	42.417	12.100	63597

Country	City	Latitude (° N)	Longitude (° E)	Population
Malta	Dingli	35.861	14.382	3347
Netherlands	Voorschoten	52.133	4.450	22783
Portugal	Almada	38.900	-9.000	101500
Spain	Albatera	38.183	-0.867	11656
Spain	Benicassim	40.050	0.067	18098
Spain	Bilbao	43.258	-2.914	875552
Spain	Cangas de Onis	43.350	-5.117	6731
Spain	Castro-Urdiales	43.373	-3.214	32258
Spain	Granada	37.183	-3.600	237929
Spain	Hostalric	41.750	2.633	3998
Spain	Jaen	37.767	-3.783	120021
Spain	Leon	42.600	-5.567	206011
Spain	Medina del Campo	41.300	-4.917	21540
Spain	Toledo	39.867	-4.017	82489
Spain	Torredembarra	41.150	1.400	15406
Spain	Valdepeñas	38.767	-3.383	31147
Spain	Velez Rubio	37.650	-2.067	7138
Spain	Zarautz	43.281	-2.168	22812
Switzerland	Brugg	47.117	7.283	10408
Switzerland	Schaffhausen	47.700	8.633	34943
Switzerland	Wallisellen	47.477	8.583	13616
Switzerland	Zurich	47.367	8.550	372047

All population data from Wikipedia.org
